# Recent advances progress of targeted drugs combined with radiotherapy for advanced non-small cell lung cancer: a review

**DOI:** 10.3389/fonc.2023.1285593

**Published:** 2023-12-05

**Authors:** Jiamin Xu, Zhongming Wang

**Affiliations:** ^1^ School of Health Science and Engineering, University of Shanghai for Science and Technology, Shanghai, China; ^2^ Department of Oncology and Radiotherapy, Shidong Hospital, Shidong Hospital Affiliated to University of Shanghai for Science and Technology, Shanghai, China

**Keywords:** targeted therapy, non-small cell lung cancer, tyrosine kinase inhibitors, whole brain radiotherapy, monoclonal antibody

## Abstract

Targeted drug therapy plays an important role in the clinical application of non-small cell lung cancer, especially adenocarcinoma. However, for patients with advanced disease, drug resistance after targeted therapy, unclear target, and other reasons that cannot or do not want surgery, the combination of chemotherapy, radiotherapy, immunity, etc. is often used. The synergistic effect of targeted drugs and radiotherapy in non-small cell lung cancer has shown good clinical efficacy. This article reviews the clinical progress of targeted drug therapy combined with radiotherapy in advanced non-small cell lung cancer in recent years, in order to provide new ideas for further clinical research of this treatment mode.

## 1 Introduction

The mortality rate of lung cancer ranks first in the world and is the main cause of cancer death worldwide (1). According to the data released by the National Cancer Center, the incidence rate and mortality of lung cancer in China are on the rise (2). Lung cancer can be roughly divided into non-small cell lung cancer (NSCLC) and small cell lung cancer (SCLC), with NSCLC accounting for approximately 80% to 85%. Since most patients are already in advanced stage (stage III or IV) when they are diagnosed, targeted therapy becomes a good choice for patients who are unwilling or unable to undergo surgery (3, 4). Targeted therapy mainly uses targeted drugs to interfere with or block specific genes, proteins or molecules that drive the occurrence and development of cancer, promote tumor cell apoptosis, and prevent cancer cell growth.

At present, NSCLC targets have been found, including epidermal growth factor receptor (EGFR), anaplastic lymphoma kinase (ALK), receptor tyrosine kinase ROS proto-oncogene 1 (ROS1) and Kirsten rat sarcoma virus oncogene (KRAS). A series of targeted drugs have been developed for different targets, such as small molecule tyrosine kinase inhibitor (TKI), monoclonal antibodies and anti-angiogenic drugs (5). Targeted therapy can improve the prognosis of NSCLC patients with or without driving mutations, with advantages such as low side effects, high accuracy, and convenient medication. However, unpredictable drug resistance limits its clinical efficacy (6). For patients with drug resistance, in addition to using new generation inhibitors or combination therapy, radiotherapy may also overcome or delay targeted drug resistance (7–10). Radiotherapy, as a palliative treatment for patients with late stage recurrent and metastatic tumors, has a wide range of applications, accurate ejaculation, and relatively small side effects. However, it is also affected by the radiosensitivity of tumor cells, and some targeted drugs can serve as radiosensitizers to improve the efficacy of radiotherapy (11–14). The synergistic effect between targeted drugs and radiotherapy makes it possible for the combination of targeted drugs and radiotherapy to achieve ideal therapeutic effects, improving patient quality of life while reducing adverse reactions.

This article reviews the clinical progress of targeted drug therapy combined with radiotherapy in advanced non-small cell lung cancer in recent years, in order to provide new ideas for further clinical research of this treatment mode.

## 2 EGFR-TKI combined with radiotherapy

### 2.1 First generation EGFR-TKI

The first generation of EGFR-TKI includes Gefitinib, Erlotinib, Icotinib, etc. Compared with single drug first-line treatment or simultaneous radiotherapy and chemotherapy, EGFR-TKI combined with radiotherapy can bring better survival benefits to patients with advanced NSCLC, especially patients with EGFR susceptibility mutation (**Table 1**).

**Table 1:** Clinical study of the first generation EGFR-TKI combined with radiotherapy for EGFR mutated NSCLC.

Researc-hers	Type	N	Methods	Clinical results					
				ORR	DCR	mOS (m)	mPFS(m)	PFS rate	OS rate
Unresectable locally advanced NSCLC									
Akamatsu et al. (11)	phase II, single arm	27	Gefitinib + Chest RT	81.5%	——	61.1	18.6	——	——
Xu et al. (12)	retrospective analysis	20	EGFR-TKI + RT	——	——	49.7	27.9	——	——
Fu et al. (13)	phase II, single arm	28	Gefitinib + Chest RT	75%	92.9%	26	11	9.5% (5 y)	30.1% (5 y)
Hotta et al. (14)	phase II, single arm	20	Gefitinib + CCRT	85%	——	——	——	90.0% (2 y)	36.9% (2 y)
Xing et al. (15)	phase II, multicenter	252	Erlotinib + RT/CCRT	70% vs 61.9%	——	——	24.5 vs 9	——	——
Advanced NSCLC									
Zheng et al. (16)	phase II, single arm	10	EGFR-TKI+ Chest RT (first line)	50%	100%	——	13	57.1% (1 y)	——
Kotek Sedef et al. (17)	retrospective analysis	141	EGFR-TKI +SBRT/EGFR-TKI+3D-CRT	——	——	46 vs 26	——	——	——
Peng et al. (18)	phase II, control test	62	EGFR-TKI+SBRT/only EGFR-TKI	——	——	33.6 vs 23.2	17.6 vs 9	——	——
Wei et al. (19)	Clinical trials	79	EGFR-TKI+ SBRT (at maximum remission/after oligo progression)	——	——	46.6 vs 51.3*	22.3 vs 12.9	——	——
Wang et al. (20)	phase III	133	EGFR-TKI+ RT/only EGFR-TKI (Less transfer)	——	——	25.5 vs 17.6	20.2 vs 12.5	99.1% vs 95.2%*(6 m)	——
Yang et al. (21)	phase III, multicenter	224	Erlotinib+ WBRT/only WBRT(BM≥2)	——	——	17.5 vs 16.9*	8.8 vs 6.4*	——	——

*, The difference was not statistically significant; m, months; y, years; ORR, objective response rate; DCR, disease control rate; mOS, median overall survival; mPFS, median progression free survival; RT, radiotherapy; CCRT, concurrent chemoradiotherapy; SBRT, stereotactic body radiation therapy; 3D-CRT, three-dimensional conformal radiotherapy; WBRT, whole brain radiotherapy; BM, brain metastases.

The meaning of "--" means "None", which is not clearly pointed out or involved in the literature.

For locally advanced NSCLC patients with EGFR sensitive mutations, EGFR-TKI combined with radiotherapy has demonstrated excellent clinical efficacy and safety. A multi-institution single arm phase II trial showed that the median progression free survival (PFS) of Gefitinib (250mg/d) combined with chest radiation therapy for locally advanced NSCLC patients with EGFR sensitive mutations was 18.6 months, the objective response rate (ORR) was 81.5%, and the overall survival (OS) was 61.1 months. The 2-year PFS rates analyzed by Wilson’s method and Kaplan Meier’s method were 29.6% and 33.3%, respectively. The incidence of grade 1 or grade 2 pneumonia was 59% and 30%, and there was no occurrence of grade 3 or above radiation pneumonia (15). The retrospective study conducted by Xu et al. and a single arm phase II experiment both yielded similar results (16, 17). On the basis of Gefitinib (250mg/d) combined with radiotherapy, synchronous chemotherapy can further improve the survival rate of locally advanced NSCLC patients with EGFR sensitive mutations. In a prospective phase II trial (LOGIK0902/OLCSG0905) (18), the 2-year OS rate of Gefitinib combined with concurrent radiotherapy and chemotherapy was 90.0%, and the 1-year and 2-year PFS rates were 58.1% and 36.9%, respectively. Throughout the treatment phase, the ORR was 85%, and no radiation pneumonia of grade 3 or above was observed. Meanwhile, compared to synchronous radiotherapy and chemotherapy, EGFR-TKI combined with radiotherapy has a better effect. In a multicenter phase II trial (19), the median PFS of Erlotinib synchronous radiotherapy was 15.5 months longer than synchronous radiotherapy and chemotherapy (24.5 months vs 9.0 months, hazard ratio [HR]=0.104, P<0.001), with significant survival benefits and an increase in ORR (70% vs 61.9%). The incidence of adverse events (AE) was similar in both groups (88.9% and 84.2%), and safety was controllable.

For patients with stage IV NSCLC carrying EGFR active mutations, a single arm phase II clinical trial (NCT02353741) showed that patients received EGFR-TKI (Erlotinib 150mg/d or Gefitinib 250mg/d) and chest radiotherapy (54-60Gy/27-30 F/5.5-6 weeks) within 2 weeks after starting EGFR-TKI treatment, with a 1-year PFS rate of 57.1% and a median PFS of 13 months, both higher than Erlotinib monotherapy (43% and 11 months). The median time to progression (TTP) of radiation disease is 20.5 months; ORR is 50%; disease control rate (DCR) is 100%; and toxicity is acceptable (10% of patients develop rash; 20% of patients develop radiation pneumonia) (20). A retrospective study reached similar conclusions and also found that patients receiving stereotactic body radiation therapy (SBRT) had better OS compared to three-dimensional conformal radiotherapy (46 months vs 26 months, P=0.05) (21). A prospective phase II Randomized controlled trial (NCT03595644) using SBRT combined with EGFR-TKI found that compared with EGFR-TKI alone, it prolonged the PFS (17.6 months vs 9.0 months, HR=0.52, P=0.016) and OS (33.6 months vs 23.2 months, HR=0.53, P=0.026) of patients, and reduced the incidence of grade 2 AE (45.2% vs 50%). And SBRT combined with EGFR-TKI did not observe toxicity of level 3 or above, and the safety is controllable (22). The clinical trial conducted by Wei et al. (23) on the timing of radiotherapy for advanced non oligometastatic NSCLC patients with EGFR mutations treated with EGFR-TKI combined with SBRT showed that primary tumor SBRT treatment at EGFR-TKI maximum remission was significantly better than primary tumor SBRT treatment after oligo progression, significantly improving PFS (22.3 months vs 12.9 months, P=0.0031), and there was no significant difference in median OS (46.6 months vs 51.3 months, P=0.54), There is no toxicity level 3 or above, and the safety is controllable.

For NCSLC patients with EGFR mutations and minimal metastasis, EGFR-TKI combined with radiotherapy also demonstrated encouraging clinical results. A phase III clinical SINDAS trial in China (NCT02893332) (24) enrolled 133 patients, including 65 patients receiving only EGFR-TKI treatment and 68 patients receiving EGFR-TKI combined radiation therapy. All patients received Gefitinib (250 mg once daily), Erlotinib (150 mg once daily), or Icotinib (25 mg three times daily) at a radiation dose of 25-40Gy/5f, EGFR-TKI combined with radiotherapy extended the median PFS by 7.7 months (12.5 months vs 20.2 months, HR=0.22, P<0.001) and median OS by 7.9 months (17.6 months vs 25.5 months, HR=0.44, P<0.001) compared to EGFR-TKI alone. The 6-month PFS of the two groups were 95.2% and 99.1%, respectively. In the EGFR-TKI combined radiotherapy group, there was no grade 5 AE, and only 7.4% of patients developed grade 3-4 pneumonia, with controllable safety.

For NSCLC patients with two or more brain metastases, the efficacy of EGFR-TKI combined with radiotherapy is not significant. A multicenter phase III clinical trial in China, ENTER (NCT01887795) (25), randomly assigned 224 individuals (1:1) to either the whole brain radiotherapy (WBRT) group alone (n=115) or the WBRT combined with Erlotinib group (n=109). The intracranial PFS of the two groups was 12.8 months and 14.6 months, respectively, with PFS of 6.4 months and 8.8 months, and OS of 16.9 months and 17.5 months. Although Erlotinib combined with WBRT did not significantly improve intracranial PFS, PSF, and OS in patients, it was safe, controllable, and well tolerated.

### 2.2 Second generation EGFR-TKI

In recent years, there are few reports on the second-generation EGFR-TKI combined with radiotherapy in the treatment of advanced NSCLC, mainly Afatinib.

A study found that Afatinib can enhance the radiosensitivity of NSCLC cells by regulating the sensitivity of cells to apoptosis (26). A retrospective study showed that (27), for NSCLC patients with EGFR mutations and brain metastases, there was no significant difference in OS between first-line Afatinib treated with brain radiation therapy (GKS or WBRT) and Afatinib alone (35.6 months vs 31.4 months, P=0.58), which was similar to the results of another previous retrospective study on Afatinib combined with WBRT compared with Afatinib alone in patients with brain metastasis of EGFR mutant lung adenocarcinoma (28). However, compared to first-line Gefitinib or Erlotinib alone, combined with brain radiation therapy significantly improved OS (41.1 months vs 25.8 months, P=0.02) (27). From this, it can be seen that Afatinib combined with brain radiation therapy may replace the first-generation EGFR-TKI single drug first-line treatment and become a new choice for NSCLC patients with EGFR mutations and brain metastases. In another retrospective study, Afatinib combined with stereotactic radiosurgery (SRS) can significantly improve the OS of patients with EGFR mutant NSCLC (lung mol GPA ≥ 3) with brain metastasis (29). In conclusion, the efficacy of Afatinib combined with intracranial radiotherapy needs to be further verified by special clinical trials.

### 2.3 Third generation EGFR-TKI

The third generation EGFR-TKI includes Osimertinib, Lazertinib, and Aumolertinib.

There is currently controversy over the efficacy of Osimertinib combined with brain radiotherapy in the treatment of NSCLC patients with EGFR mutations and brain metastases. A retrospective study showed that the combination of Osimertinib treatment and brain radiation therapy did not significantly improve patient survival. Compared with Osimertinib treatment alone, there was no significant difference in patient progression time (8.5 months vs 6.9 months, P=0.13), intracranial progression time (14.8 months vs 20.5 months, P=0.51), and TTF (13.8 months vs 8.6 months, P=0.26) (30). Another retrospective analysis showed encouraging results, which found that brain radiation therapy before disease progression to Osimertinib treatment can prolong intracranial progression time and demonstrate excellent PFS and OS (31). It can be seen that the clinical efficacy of this treatment mode requires further validation through prospective clinical trials. The efficacy of Osimertinib combined with radiotherapy varies depending on the type of EGFR mutation in NSCLC patients. A retrospective study showed that the combination of Osimertinib and brain radiation therapy can bring survival benefits to L858R mutation patients (median OS was 18.8 months and 29.9 months, respectively), but for 19DEL mutation patients, Osimertinib combined with radiation therapy is less effective than Osimertinib alone. It is worth noting that combination therapy may lead to white matter encephalopathy (32). To evaluate the clinical efficacy and safety of Osimertinib after radiotherapy and chemotherapy in locally advanced NSCLC patients with positive EGFR mutations, Lu et al. (33) conducted a global multicenter phase III LAURA trial (NCT03521154), which is currently ongoing.

The efficacy of Lazertinib combined with radiotherapy is not yet known, and two clinical trials are currently being conducted in South Korea, and the results may provide valuable information for clinical research. A prospective multicenter single arm phase II clinical trial (NCT05338619) was conducted to evaluate the clinical efficacy and safety of Lazertinib as consolidation therapy for unresectable EGFR mutated positive NSCLC patients after synchronous radiotherapy and chemotherapy (34). Another multicenter dual arm phase II trial (NCT05167851) aims to investigate the clinical efficacy and safety of Lazertinib combined with early local ablation radiotherapy in patients with EGFR mutated NSCLC with simultaneous oligometastatic disease (35). Both trials targeted EGFR mutation positive patients, while there is currently no research on the effectiveness of Lazertinib combined with radiotherapy for negative patients.

Similarly, the efficacy of Aumolertinib combined with radiotherapy in advanced NSCLC patients is not yet clear. To investigate the safety of Aumolertinib combined with radiotherapy for locally advanced NSCLC patients with EGFR mutations, Zhu et al. (36) conducted a multicenter phase II study (NCT04636593), and the results are expected.

In conclusion, EGFR-TKI combined with radiotherapy has great potential in the treatment of advanced NSCLC, while for the incidence of radiation pneumonia in patients with advanced Adenocarcinoma of the lung treated by the first generation, second generation or third generation EGFR-TKI combined with chest radiotherapy, a study made a clinical and imaging comparison, and found that the incidence of radiation pneumonia in the first generation or third generation EGFR-TKI combined with chest radiotherapy was lower than that in the second generation EGFR-TKI combined with chest radiotherapy. The overall incidence of clinical radiation pneumonia in the three groups was 29%, 48%, and 28%, respectively (P=0.043), while the overall incidence of imaging radiation pneumonia was 33%, 58%, and 36%, respectively (P=0.010) (37). Another retrospective study compared the incidence of grade 2 and above radiation pneumonia in first-generation EGFR-TKI (Gefitinib, Erlotinib, and Icotinib) synchronous chest radiotherapy. It was found that although the incidence of ≥ grade 2 radiation pneumonia was higher than that of synchronous radiotherapy and chemotherapy, the use of Icotinib, ipsilateral lung V30 ≤ 34%, or EGFR-TKI overlapping with chest radiotherapy for ≤ 20 days can be reduced (38). Although the optimal timing of EGFR-TKI combined with radiotherapy is not yet clear, and the specific treatment plan still needs to be optimized according to the patient’s own situation, it is worth noting that early radiotherapy for the primary tumor in the process of combined treatment will have better curative effect and is more likely to improve the prognosis of NCSLC patients.

## 3 ALK/ROS1-TKI combined with radiotherapy

Crizotinib and Lorlatinib belong to multi-target TKIs, which can effectively target tumours with ALK or ROS1 gene fusion mutations. For untreated patients with ALK positive NSCLC, the efficacy of Lorlatinib is more durable and significant than Crizotinib. In the crown phase 3 study, the 3-year PFS was 64% and 19%, respectively (39). Lorlatinib also showed good intracranial activity in patients with ros1 rearranged NSCLC who developed CNS progression after Crizotinib treatment (40). At present, the efficacy of ALK/ROS1-TKI combined with radiotherapy is not clear, and most of them are for NSCLC patients with brain metastasis. In the Alex study, the efficacy of Crizotinib in patients with ALK positive NSCLC who received previous radiotherapy was better than that of untreated patients, and the ORR of the central nervous system was 71.4% and 40.0%, respectively. The same was true for Alectinib (a highly effective and selective ALK-TKI), which was higher than Crizotinib, and the ORR of the central nervous system was 85.7% and 78.6%, respectively (41). Tae684 is a specific ALK-TKI. A German study found for the first time that tae684 can effectively and selectively enhance the anti-proliferative and pro apoptotic effects of photon/carbon ion radiotherapy on ALK positive NSCLC (42). This result is gratifying and lays a foundation for clinical trials, which is worthy of further study. The AE produced by combination therapy cannot be ignored. Different from the previous sequential administration, a Japanese team administered ALK-TKI (Crizotinib/Alectinib/Ceritinib) and WBRT simultaneously to patients with brain metastasis of ALK rearranged NSCLC for the first time, and found that this method may cause serious ototoxicity (43).

## 4 KRAS inhibitor combined with radiotherapy

In recent years, breakthrough progress has been made in targeted drug research for KRAS mutations. Sotorasib and Adagrasib have broken the claim that KRAS is a “ no drug” target, bringing good news to KRAS mutated tumor patients.

For KRAS G12C mutant NSCLC patients who have previously received chemotherapy or immune checkpoint inhibitors, the use of Sotorasib in the CodeBreakK 200 study showed significant survival benefits compared to standard treatment (Docetaxel), with median PFS of 4.5 months and 5.6 months, respectively (p=0.0017) (44). In the KRYSTAL-1 Phase 1-2 study, the median PFS and median OS of Adagrasib treatment in these patients were 6.5 months and 12.6 months respectively, and the efficacy was also surprising (45). It is worth noting that there are currently no clinical studies on the combination of KRAS inhibitors and radiotherapy for the treatment of NSCLC. However, in the KRAS mutated mouse model, the use of Sotorasib can reduce the increased expression of radiation induced programmed death ligand 1, demonstrating encouraging anti-tumor activity (46). From this, it can be seen that for patients with advanced NSCLC, KRAS inhibitors combined with radiotherapy is a promising strategy that deserves further clinical research.

## 5 MEK inhibitors combined with radiotherapy

MEK protein is a mitogen activated extracellular signal-regulated kinase that participates in the regulation of numerous processes such as cell proliferation, differentiation, metabolism, and apoptosis. The RAS/RAF/MEK/ERK signaling pathway is overexpressed or mutated in many malignant tumors, while MEK inhibitors can block tumor cell proliferation and induce cell death by inhibiting downstream signaling (47). Currently, research on the combination of MEK inhibitors and radiotherapy in advanced NSCLC patients is mainly focused on Trametinib and Selumetinib. Some studies have found that (46), MEK inhibitors combined with radiotherapy can increase the expression of major histocompatibility complex class I on the surface of tumor cells, activate anti-cancer immunity *in vivo*, improve the ability of T cells to recognize and kill cancer cells, so as to achieve better anti-tumor effect.

In the Phase I clinical trial (NCT01912625), the combination of Trametinib and synchronous radiotherapy and chemotherapy showed good clinical efficacy (48). For non-metastatic locally advanced NSCLC patients with KRAS mutations, the median PFS and median OS of Trametinib (1.5mg) combined with concurrent radiotherapy and chemotherapy were 11 months and 38 months, respectively, with controllable safety. The results of Selumetinib combined with radiotherapy were opposite. In a single arm, single center phase I trial (NCT01146756), Selumetinib combined with chest radiotherapy was used to treat stage III or stage IV NSCLC patients with chest symptoms. Patients had poor prognosis and severe lymphocyte depletion, with PFS of 23.8% and 9.5% at one year and two years, respectively, and median OS and PSF of 9.7 and 6.9 months, respectively (49).

The combination of Trametinib and synchronous radiotherapy and chemotherapy has achieved good results in the treatment of advanced NSCLC, but for patients who are not suitable for synchronous radiotherapy and chemotherapy, the results of Selumetinib combined radiotherapy are not satisfactory. The efficacy and safety of Trametinib combined radiotherapy still need further clinical trials to verify.

## 6 Monoclonal antibody combined with radiotherapy

Nimotuzumab and Cetuximab are humanized IgG1 monoclonal EGFR antibodies. Nimotuzumab combined with concurrent radiotherapy and chemotherapy shows excellent clinical efficacy in the treatment of advanced NSCLC, especially squamous cell lung cancer, while Cetuximab combined with concurrent radiotherapy and chemotherapy will show different results due to different chemotherapy drugs. It is worth noting that the current research focuses on patients with unresectable locally advanced NSCLC.

### 6.1 Nimotuzumab

In the Japanese multicenter single arm phase II trial (JapicCTI-090825), Nimotuzumab combined with synchronous radiotherapy and chemotherapy for unresectable locally advanced NSCLC patients had a treatment completion rate of 87.2%, an ORR of 69.2%, good tolerance, no rash or radiation pneumonia of grade 3 or above, a median PFS of 508 days, and a 5-year PFS rate and OS rate of 29% and 58.4%, respectively, demonstrating excellent clinical efficacy, Especially in patients with squamous cell lung cancer (with a 5-year PFS rate of 50%) (50). In a prospective phase II randomized trial, Qiu et al. evaluated the impact of adding Nimotuzumab during synchronous radiotherapy and chemotherapy on clinical outcomes in squamous cell lung cancer. The study found that compared with synchronous radiotherapy and chemotherapy alone, there was no significant difference in median OS (24.9 months vs 23.5 months) and median PFS (12.1 months vs 13.7 months), but Nimotuzumab combined with synchronous radiotherapy and chemotherapy showed good tolerance and reduced risk of brain metastasis (51).

### 6.2 Cetuximab

For unresectable stage III NSCLC patients, the RTOG 0617 study showed that adding Cetuximab to standard radiation dose (60Gy) synchronous chemotherapy (carboplatin and paclitaxel) did not bring survival benefits. The median OS with or without Cetuximab was 2 years, with a 5-year OS rate of 32.1% (52). However, another phase II trial (IFCT-0803) showed that Cetuximab combined with concurrent radiotherapy and chemotherapy (cisplatin and pemetrexed) was feasible and clinically active, with an ORR of 90.5% at week 16, one-year and two-year survival rates of 75.8% and 59.5%, respectively. The median OS was 35.8 months, the median PFS was 14.4 months, and the one-year and two-year PFS were 57.6% and 34.3%, respectively (53). It can be seen that the clinical efficacy of Cetuximab combined with synchronous radiotherapy and chemotherapy is still unclear.

## 7 Antiangiogenic drugs combined with radiotherapy

### 7.1 Recombinant human endostatin

Recombinant human endostatin (Rh-endostatin) is a novel anti angiogenic drug developed in China. Some studies have shown that Rh-endostatin can reduce the increased CXCR4 expression involved in the recruitment of Tumor-associated macrophage due to radiotherapy, promote the normalization of tumor blood vessels, and thus enhance the radiotherapy effect (54). A series of clinical studies have been conducted on Rh-endostatin combined with radiotherapy in advanced NSCLC (**Table 2**).

**Table 2:** Clinical study of Rh-endostatin combined with radiotherapy in advanced NSCLC.

Researchers	Research type	N	Patient type	Methods	Clinical results			
					mOS (m)	mPFS(m)	PFS rate	OS rate
Zhu et al. (40)	retrospective analysis	—	unresectable stages III and IV	Only RT/Rh-endostatin +RT	13.1 vs 40.0	4.4 vs 8.0	—	—
Zhai et al. (41)	phase II	67	unresectable stages III	Continuous intravenous injection Rh-endostatin +CCRT	34.7	13.3	34.8% (2 y)	59.9% (2 y)
Ma et al. (42)	update follow-up of two phase II trials	115	unresectable stages III	Continuous intravenous injection Rh-endostatin+ CCRT/Continuous venous pumping Rh-endostatin + CCRT	38.5 vs 24.0*	15.4 vs 9.9*	27.7% vs 18.3% (5 y)	41.0% vs 31.1%(5 y)
Chen et al. (43)	randomized controlled test	43	brain metastases	Rh-endostatin +WBRT/only WBRT	14.2 vs 6.4	8.1 vs 4.9	—	—

*, The difference was not statistically significant; mOS, median overall survival; mPFS, median progression free survival; RT, radiotherapy; CCRT, concurrent chemoradiotherapy; WBRT, whole brain radiation therapy; m, months; y, years.

The meaning of "--" means "None", which is not clearly pointed out or involved in the literature.

For unresectable patients with advanced NSCLC, a retrospective study showed that compared with radiotherapy alone, Rh-endostatin combined with radiotherapy reduced the recurrence rate of radiation pneumonia, mortality rate of radiation pneumonia and Pulmonary fibrosis rate, improved the median PFS by 3.6 months (8.0 months vs 4.4 months, HR=0.53, P=0.019), and significantly prolonged the median OS, which was 40.0 months vs 13.1 months (HR=0.53, P=0.045) (55). The combination of Rh-endostatin and radiotherapy and chemotherapy also achieved good results. The prospective Phase II HELPER trial showed that although continuous intravenous injection of Rh-endostatin combined with synchronous radiotherapy and chemotherapy did not significantly prolong the median PFS (12 months vs 13.3 months), it achieved better OS (34.7 months) and good distant metastasis free survival (41.7 months), with the hope of achieving 2-year PFS and tolerable toxicity (56). In addition, different Route of administration of Rh-endostatin may also affect the efficacy and safety of locally advanced NSCLC patients. A study compared the NCT01218594 and NCT01733589 trials and found that when Rh-endostatin was combined with synchronous radiotherapy and chemotherapy, continuous intravenous pumping achieved better survival rates than intravenous injection. The median PFS was 15.4 months and 9.9 months (HR=0.751, P=0.200), the median OS was 38.5 months and 24.0 months (HR=0.746, P=0.209), and the five-year PFS and OS rates were 27.7% vs 18.3% and 41.0% vs 31.1%, respectively. Moreover, it has reduced the incidence of adverse blood reactions (such as leukopenia, lymphopenia, etc.) (57).

In addition, Rh-endostatin combined with radiotherapy can also significantly improve the survival rate of NSCLC patients with brain metastases. In the Randomized controlled trial (NCT03614065) carried out by Chen et al. (58), 43 NSCLC patients with brain metastases were randomized to receive Rh-endostatin combined with WBRT (n=19) and WBRT alone (n=24). The median PFS of the two groups were 8.1 months and 4.9 months, the intracranial PFS were 11.6 months and 4.8 months, and the OS were 14.2 months and 6.4 months, respectively. There were no severe AEs observed in the experiment, and most AEs were well tolerated.

### 7.2 Anlotinib

Anlotinib is a new multi target Tyrosine kinase inhibitor, targeting Vascular endothelial growth factor receptor (VEFGR) 2 and 3, Fibroblast growth factor receptor factor receptor 1-4, and platelet-derived growth factor receptor α and β,C-Kit and Ret, thereby inhibiting tumor growth and angiogenesis (59). Studies have found that Anlotinib activates cGAS/STING signals in NSCLC, promoting the infiltration and activation of CD8+T cells stimulated by ionizing radiation, thereby enhancing radiation sensitivity (60). Anlotinib combined with radiotherapy conducted a series of clinical studies (**Table 3**).

**Table 3:** Clinical study of Anlotinib combined with radiotherapy in advanced NSCLC.

Researchers	Research type	N	Patient type	Methods	Clinical results			
					iORR	mOS (m)	miPFS (m)	Relief rate of intracranial hypertension
Wang et al. (46)	retrospective analysis	46	BM	Anlotinib +SRS/only SRS	80.9% vs 60.0%*	——	13.9 vs 11.4	71.4% vs 12%
He et al. (47)	retrospective analysis	74	No mutation, BM	Anlotinib + RT/only RT	——	8.5 vs 6.0*	11.0 vs 3.0	——
Liu et al. (48)	phase II, single arm	21	No mutation, multiple BM	Anlotinib +WBRT	66.7%	13.4	10.3	——

*, The difference was not statistically significant; m, months; BM, brain metastases; iORR, intracranial objective response rate; mOS, median overall survival; miPFS, median intracranial progression free survival; RT, radiotherapy; SRS, stereotactic radiosurgery; WBRT, whole brain radiation therapy.

The meaning of "--" means "None", which is not clearly pointed out or involved in the literature.

Wang et al. (61) carried out a retrospective Cohort study to explore the efficacy and safety of Anlotinib combined with stereotactic radiosurgery (SRS) in the treatment of brain metastases from non-small cell lung cancer. 46 patients with brain metastases from NSCLC were divided into a combined treatment group (n=21) and a single SRS group (n=25) according to the different treatment methods, and found that the combined treatment showed gratifying results, The remission rate of intracranial hypertension was higher in the SRS group alone (71.4% vs 12.0%, P<0.001), while the incidence of radiation induced brain necrosis was lower in the SRS group alone (3% vs 20%, P=0.030). The intracranial PFS of the two groups was 13.9 ± 2.4 months and 11.4 ± 1.8 months, respectively (P<0.001). The incidence of drug-related adverse reactions in the combination therapy group was 9.5%, and the safety was controllable. For patients with NSCLC brain metastasis without driver mutations, a retrospective analysis found that the combination of Anlotinib and brain radiation therapy can improve the survival rate of NSCLC patients. Compared with brain radiation therapy alone, the combination therapy prolonged the patient’s intracranial PFS (3.0 months vs 11.0 months, P=0.048) (62). A single arm phase II study (ChiCTR 1900027769) further validated the above results. Anlotinib combined with WBRT showed good clinical efficacy and tolerability in NSCLC patients without driver mutations and with multiple brain metastases. Intracranial PFS and OS were 10.3 months and 13.4 months, respectively. DCR for intracerebral and extracerebral lesions were 90.5% and 81.0%, respectively, and no severe AE occurred (63).

From this, it can be seen that Anlotinib combined with brain radiotherapy (SRS or WBRT) can bring significant survival benefits to NSCLC patients with brain metastasis, providing an additional clinical option for patients who cannot receive concurrent radiotherapy and chemotherapy.

### 7.3 Apatinib

Apatinib is a Tyrosine kinase inhibitor that can selectively inhibit VEGFR-2. A study found that low-dose Apatinib (60mg/kg) can promote the normalization of tumor blood vessels and significantly relieve intratumoral hypoxia, thereby enhancing the radiosensitivity of Lewis lung cancer xenograft mice (64). In addition, Apatinib can also enhance radiosensitivity by inhibiting DNA double strand break repair caused by radiation and downregulating AKT and ERK signaling in NSCLC cells (65). The combination of Apatinib and WBRT in the treatment of symptomatic NSCLC patients with multiple brain metastases and peritumoral brain edema resulted in an intracranial ORR of 84.6% and a median intracranial PFS of 6.97 months, both of which were superior to chemotherapy combined with WBRT (intracranial ORR of 47.6% and median intracranial PFS of 4.77 months). No grade 3 or 4 AE was observed, indicating good tolerance and controllable safety. But the median OS of the two groups was similar, at 7.70 months and 6.67 months, respectively (66). This study has certain limitations due to its small sample size and retrospective characteristics. The multi-center Phase II open Randomized controlled trial (NCT03801200) carried out by Ma et al. is in progress, and its results may verify the above conclusions and expand the treatment options of this population (67).

## 8 Conclusion

Non-small cell lung cancer has entered the era of precision treatment, and the targeted combination radiotherapy treatment model has the potential to improve the survival rate of non-small cell lung cancer patients. For locally advanced, advanced, or non-small cell lung cancer with brain metastasis, regardless of whether it has driving mutations, this mode has its therapeutic advantages. However, the selection of targeted drugs, dosage and Route of administration, selection of radiotherapy technology, radiation dose, radiation timing, and toxicity caused by combined treatment are all issues that need to be considered in clinical treatment, and more clinical studies are needed to find appropriate treatment plans. With the in-depth research of targeted drugs and the continuous optimization of radiotherapy technology, Targeted therapy combined with radiotherapy for advanced non-small cell lung cancer can achieve better results, which is expected to improve the prognosis of patients and improve the survival rate of patients. In addition, immunotherapy is emerging, including immune checkpoint inhibitors (such as Ipilimumab, Nivolumab, Durvalumab, Pembrolizumab, Tremelimumab, etc.), cancer vaccines, bispecific antibodies, adoptive cell therapy, etc. combined with targeted drugs or radiotherapy may bring new hope to patients with advanced non-small cell lung cancer, which is worthy of further attention and research.

## Author contributions

JX: Writing – original draft, Writing – review and editing. ZW: Writing – review and editing.

## References

[1] Sung H Ferlay J Siegel RL Laversanne M Soerjomataram I Jemal A. Global cancer statistics 2020: GLOBOCAN estimates of incidence and mortality worldwide for 36 cancers in 185 countries. CA Cancer J Clin (2021) 71:209–49. doi: 10.3322/caac.21492 33538338

[2] Zheng R Zhang S Wang S Chen R Sun K Zeng H. Lung cancer incidence and mortality in China: Updated statistics and an overview of temporal trends from 2000 to 2016. J Natl Cancer Center (2022) 2:139–47. doi: 10.1016/j.jncc.2022.07.004 PMC11256716 39036448

[3] Ettinger DS Wood DE Aisner DL Akerley W Bauman JR Bharat A. NCCN guidelines® Insights: non–small cell lung cancer, version 2.2023: featured updates to the NCCN guidelines. J Natl Compr Canc Netw (2023) 21:340–50. doi: 10.6004/jnccn.2023.0020 37015337

[4] Shi Y Chen G Wang X Liu Y Wu L Hao Y. Furmonertinib (AST2818) versus gefitinib as first-line therapy for Chinese patients with locally advanced or metastatic EGFR mutation-positive non-small-cell lung cancer (FURLONG): a multicentre, double-blind, randomised phase 3 study. Lancet Respir Med (2022) 10:1019–28. doi: 10.1016/S2213-2600(22)00168-0 35662408

[5] Wu J Lin Z. Non-small cell lung cancer targeted therapy: drugs and mechanisms of drug resistance. Int J Mol Sci (2022) 23:15056. doi: 10.3390/ijms232315056 36499382 PMC9738331

[6] Yu W Ye F Yuan X Ma Y Mao C Li X. A phase I/II clinical trial on the efficacy and safety of NKT cells combined with gefitinib for advanced EGFR-mutated non-small-cell lung cancer. BMC Cancer (2021) 21:877. doi: 10.1186/s12885-021-08590-1 34332557 PMC8325186

[7] Chicas-Sett R Castilla Martinez J Hernández Blanquisett A Zafra J Pastor-Peidro J. Stereotactic ablative radiotherapy for acquired resistance to EGFR therapy in metastatic non-small cell lung cancer. Front Oncol (2023) 12:1092875. doi: 10.3389/fonc.2022.1092875 36727053 PMC9884815

[8] Novak J Salgia R West H Villalona-Calero MA Sampath S Williams T. Ablative radiotherapy as a strategy to overcome TKI resistance in EGFR-mutated NSCLC. Cancers (Basel) (2022) 14:3983. doi: 10.3390/cancers14163983 36010982 PMC9406789

[9] Bertino EM Gentzler RD Clifford S Kolesar J Muzikansky A Haura EB. Phase IB study of osimertinib in combination with navitoclax in EGFR-mutant NSCLC following resistance to initial EGFR therapy (ETCTN 9903). Clin Cancer Res (2021) 27:1604–11. doi: 10.1158/1078-0432.CCR-20-4084 PMC7976451 33376097

[10] Bylicki O Tomasini P Radj G Guisier F Monnet I Ricordel C. Atezolizumab with or without bevacizumab and platinum-pemetrexed in patients with stage IIIB/IV non-squamous non-small cell lung cancer with EGFR mutation, ALK rearrangement or ROS1 fusion progressing after targeted therapies: A multicentre phase II open-label non-randomised study GFPC 06-2018. Eur J Cancer (2023) 183:38–48. doi: 10.1016/j.ejca.2023.01.014 36801605

[11] Wu S Zhu L Tu L Chen S Huang H Zhang J. AZD9291 increases sensitivity to radiation in PC-9-IR cells by delaying DNA damage repair after irradiation and inducing apoptosis. Radiat Res (2018) 189:283–91. doi: 10.1667/RR14682.1 29332537

[12] Jost T Schuster B Heinzerling L Weissmann T Fietkau R Distel LV. Kinase inhibitors increase individual radiation sensitivity in normal cells of cancer patients. Strahlenther Onkol (2022) 198:838–48. doi: 10.1007/s00066-022-01945-y PMC9402507 35471558

[13] Li F Bing Z Chen W Ye F Liu Y Ding L. Prognosis biomarker and potential therapeutic target CRIP2 associated with radiosensitivity in NSCLC cells. Biochem Biophys Res Commun (2021) 584:73–9. doi: 10.1016/j.bbrc.2021.11.002 34773852

[14] Zhu X Wang Y Jiang C Li X Sun L Wang G. Radiosensitivity-specific proteomic and signaling pathway network of non-small cell lung cancer (NSCLC). Int J Radiat Oncol Biol Phys (2022) 112:529–41. doi: 10.1016/j.ijrobp.2021.08.041 34506873

[15] Akamatsu H Murakami H Harada H Shimizu J Hayashi H Daga H. Gefitinib with concurrent thoracic radiotherapy in unresectable locally advanced NSCLC with EGFR mutation; west Japan oncology group 6911L. J Thorac Oncol (2021) 16:1745–52. doi: 10.1016/j.jtho.2021.05.019 34116229

[16] Xu K Liang J Zhang T Zhou Z Chen D Feng Q. Clinical outcomes and radiation pneumonitis after concurrent EGFR-tyrosine kinase inhibitors and radiotherapy for unresectable stage III non-small cell lung cancer. Thorac Cancer (2021) 12:814–23. doi: 10.1111/1759-7714.13816 PMC7952784 33501781

[17] Fu Z Yang X Wang W Deng L Zhang T Bi N. Radiotherapy combined with gefitinib for patients with locally advanced non-small cell lung cancer who are unfit for surgery or concurrent chemoradiotherapy: a phase II clinical trial. Radiat Oncol (2020) 15:155. doi: 10.1186/s13014-020-01596-2 32563259 PMC7305585

[18] Hotta K Saeki S Yamaguchi M Harada D Bessho A Tanaka K. Gefitinib induction followed by chemoradiotherapy in EGFR-mutant, locally advanced non-small-cell lung cancer: LOGIK0902/OLCSG0905 phase II study. ESMO Open (2021) 6:100191. doi: 10.1016/j.esmoop.2021.100191 34153652 PMC8233144

[19] Xing L Wu G Wang L Li J Wang J Yuan Z. Erlotinib versus etoposide/cisplatin with radiation therapy in unresectable stage III epidermal growth factor receptor mutation-positive non-small cell lung cancer: A multicenter, randomized, open-label, phase 2 trial. Int J Radiat Oncol Biol Phys (2021) 09:1349–58. doi: 10.1016/j.ijrobp.2020.11.026 33220395

[20] Zheng L Wang Y Xu Z Yang Q Zhu G Liao XY. Concurrent EGFR-TKI and thoracic radiotherapy as first-line treatment for stage IV non-small cell lung cancer harboring EGFR active mutations. Oncologist (2019) 24:1031–e612. doi: 10.1634/theoncologist.2019-0285 31040256 PMC6693693

[21] Kotek Sedef A Akkus Yildirim B Topkan E Taner Sumbul A. Upfront thoracic radiotherapy to primary lesion improves outcomes in patients with stage IV non-small cell lung cancer harboring EGFR mutations. J BUON (2021) 26:1446–52. 34565003

[22] Peng P Gong J Zhang Y Zhou S Li Y Han G. EGFR-TKIs plus stereotactic body radiation therapy (SBRT) for stage IV Non-small cell lung cancer (NSCLC): A prospective, multicenter, randomized, controlled phase II study. Radiother Oncol (2023) 184:109681. doi: 10.1016/j.radonc.2023.109681 37105304

[23] Wei H Zhou X Yang H Gong Y Wang J Xu Y. Stereotactic body radiotherapy to the primary lung lesion improves the survival of the selected patients with non-oligometastatic NSCLC harboring EGFR activating mutation with first-line EGFR-TKIs: a real-world study. J Cancer Res Clin Oncol (2022) 148:2589–98. doi: 10.1007/s00432-021-03831-z PMC11800994 34669037

[24] Wang XS Bai YF Verma V Yu RL Tian W Ao R. Randomized trial of first-line tyrosine kinase inhibitor with or without radiotherapy for synchronous oligometastatic EGFR -mutated non-small cell lung cancer. J Natl Cancer Inst (2023) 115:742–8. doi: 10.1093/jnci/djac015 PMC10248839 35094066

[25] Yang Z Zhang Y Li R Yisikandaer A Ren B Sun J. Whole-brain radiotherapy with and without concurrent erlotinib in NSCLC with brain metastases: a multicenter, open-label, randomized, controlled phase III trial. Neuro Oncol (2021) 23:967–78. doi: 10.1093/neuonc/noaa281 PMC8168818 33331923

[26] Zhang P Song E Jiang M Song Y. Celecoxib and Afatinib synergistic enhance radiotherapy sensitivity on human non-small cell lung cancer A549 cells. Int J Radiat Biol (2021) 97:170–8. doi: 10.1080/09553002.2021.1846817 33164600

[27] Jung HA Park S Lee SH Ahn JS Ahn MJ Sun JM. The role of brain radiotherapy before first-line afatinib therapy, compared to gefitinib or erlotinib, in patients with EGFR-mutant non-small cell lung cancer. Cancer Res Treat (2023) 55:479–87. doi: 10.4143/crt.2022.1344 PMC10101777 36596729

[28] Li SH Liu CY Hsu PC Fang YF Wang CC Kao KC. Response to afatinib in treatment-naïve patients with advanced mutant epidermal growth factor receptor lung adenocarcinoma with brain metastases. Expert Rev Anticancer Ther (2018) 18:81–9. doi: 10.1080/14737140.2018.1409623 29172778

[29] Cheng WC Shen YC Chien CR Liao WC Chen CH Hsia TC. The optimal therapy strategy for epidermal growth factor receptor-mutated non-small cell lung cancer patients with brain metastasis: A real-world study from Taiwan. Thorac Cancer (2022) 13:1505–12. doi: 10.1111/1759-7714.14423 PMC9108041 35394114

[30] Thomas NJ Myall NJ Sun F Patil T Mushtaq R Yu C. Brain metastases in EGFR- and ALK-positive NSCLC: outcomes of central nervous system-penetrant tyrosine kinase inhibitors alone versus in combination with radiation. J Thorac Oncol (2022) 17:116–29. doi: 10.1016/j.jtho.2021.08.009 34455066

[31] Yu F Ni J Zeng W Zhou Y Guo T Zeng Y. Clinical value of upfront cranial radiation therapy in osimertinib-treated epidermal growth factor receptor-mutant non-small cell lung cancer with brain metastases. Int J Radiat Oncol Biol Phys (2021) 111:804–15. doi: 10.1016/j.ijrobp.2021.05.125 34058255

[32] Zhai X Li W Li J Jia W Jing W Tian Y. Therapeutic effect of osimertinib plus cranial radiotherapy compared to osimertinib alone in NSCLC patients with EGFR-activating mutations and brain metastases: a retrospective study. Radiat Oncol (2021) 16:233. doi: 10.1186/s13014-021-01955-7 34865626 PMC8647301

[33] Lu S Casarini I Kato T Cobo M Özgüroğlu M Hodge R. Osimertinib maintenance after definitive chemoradiation in patients with unresectable EGFR mutation positive stage III non-small-cell lung cancer: LAURA trial in progress. Clin Lung Cancer (2021) 22:371–5. doi: 10.1016/j.cllc.2020.11.004 33558193

[34] Choi J Lee JE Choi CM Oh IJ Lee KY Jang TW. multicenter study of lazertinib as consolidation therapy in patients with locally advanced, unresectable, EGFR mutation-positive non-small cell lung cancer (stage III) who have not progressed following definitive, platinum-based, chemoradiation therapy (PLATINUM trial). Thorac Cancer (2022) 13:3431–5. doi: 10.1111/1759-7714.14663 PMC9715807 36259253

[35] Kim KH Yoon S Ahn HK Lee SY Lee GW Lee SS. A multicenter two-arm, phase II trial assessing the safety and efficacy of first-line lazertinib and locally ablative radiotherapy in patients with synchronous oligo-metastatic EGFR-mutant non-small cell lung cancer (ABLATE, KCSG-LU21-11). Clin Lung Cancer (2022) 23:e536–9. doi: 10.1016/j.cllc.2022.07.014 36002368

[36] Zhu L Zou C Zhang Z Wang J Yang L Rao C. Thoracic radiotherapy and concurrent almonertinib for unresectable stage III EGFR-mutated non-small-cell lung cancer: a phase 2 study. BMC Cancer (2021) 21:511. doi: 10.1186/s12885-021-08266-w 33962566 PMC8103745

[37] Mu F Fan B Li B Qin W Li H Wang C. Comparison of the incidence rate of radiation pneumonitis observed in patients with advanced lung adenocarcinoma treated with simultaneous thoracic radiotherapy and 1G/2G/3G EGFR-TKIs. Cancer Manag Res (2023) 15:351–62. doi: 10.2147/CMAR.S404874 PMC10106805 37077536

[38] Jia W Gao Q Wang M Li J Jing W Yu J. Overlap time is an independent risk factor of radiation pneumonitis for patients treated with simultaneous EGFR-TKI and thoracic radiotherapy. Radiat Oncol (2021) 16:41. doi: 10.1186/s13014-021-01765-x 33622352 PMC7903606

[39] Solomon BJ Bauer TM Mok TSK Liu G Mazieres J de Marinis F. Efficacy and safety of first-line lorlatinib versus crizotinib in patients with advanced, ALK-positive non-small-cell lung cancer: updated analysis of data from the phase 3, randomised, open-label CROWN study. Lancet Respir Med (2023) 11:354–66. doi: 10.1016/S2213-2600(22)00437-4 36535300

[40] Schneider JL Muzikansky A Lin JJ Krueger EA Lennes IT Jacobson JO. A phase 2 study of lorlatinib in patients with ROS1-rearranged lung cancer with brain-only progression on crizotinib. JTO Clin Res Rep (2022) 3:100347. doi: 10.1016/j.jtocrr.2022.100347 35815322 PMC9257415

[41] Gadgeel S Peters S Mok T Shaw AT Kim DW Ou SI. Alectinib versus crizotinib in treatment-naive anaplastic lymphoma kinase-positive (ALK+) non-small-cell lung cancer: CNS efficacy results from the ALEX study. Ann Oncol (2018) 29:2214–22. doi: 10.1093/annonc/mdy405 PMC6290889 30215676

[42] Dai Y Wei Q Schwager C Hanne J Zhou C Herfarth K. Oncogene addiction and radiation oncology: effect of radiotherapy with photons and carbon ions in ALK-EML4 translocated NSCLC. Radiat Oncol (2018) 13:1. doi: 10.1186/s13014-017-0947-0 29304828 PMC5756447

[43] Nakashima T Nonoshita T Hirata H Inoue K Nagashima A Yoshitake T. Adverse events of concurrent radiotherapy and ALK inhibitors for brain metastases of ALK-rearranged lung adenocarcinoma. In Vivo (2020) 34:247–53. doi: 10.21873/invivo.11767 PMC6984098 31882485

[44] de Langen AJ Johnson ML Mazieres J Dingemans AC Mountzios G Pless M. Sotorasib versus docetaxel for previously treated non-small-cell lung cancer with KRASG12C mutation: a randomised, open-label, phase 3 trial. Lancet (2023) 401:733–46. doi: 10.1016/S0140-6736(23)00221-0 36764316

[45] Jänne PA Riely GJ Gadgeel SM Heist RS Ou SI Pacheco JM. Adagrasib in non-small-cell lung cancer harboring a KRASG12C mutation. N Engl J Med (2022) 387:120–31. doi: 10.1056/NEJMoa2204619 35658005

[46] Zheng Y Liu Y Zhang F Su C Chen X Zhang M. Radiation combined with KRAS-MEK inhibitors enhances anticancer immunity in KRAS-mutated tumor models. Transl Res (2023) 252:79–90. doi: 10.1016/j.trsl.2022.08.005 35948200

[47] Han J Liu Y Yang S Wu X Li H Wang Q. MEK inhibitors for the treatment of non-small cell lung cancer. J Hematol Oncol (2021) 14:1. doi: 10.1186/s13045-020-01025-7 33402199 PMC7786519

[48] Lin SH Lin HY Verma V Xu-Welliver M Thall PF Yao L. Phase I trial of definitive concurrent chemoradiotherapy and trametinib for KRAS-mutated non-small cell lung cancer. Cancer Treat Res Commun (2022) 30:100514. doi: 10.1016/j.ctarc.2022.100514 35051703 PMC9259763

[49] Haslett K Koh P Hudson A Ryder WD Falk S Mullan D. Phase I trial of the MEK inhibitor selumetinib in combination with thoracic radiotherapy in non-small cell lung cancer. Clin Transl Radiat Oncol (2021) 28:24–31. doi: 10.1016/j.ctro.2021.02.008 33748440 PMC7970011

[50] Yamamoto N Harada H Okamoto I Masuda N Hayakawa K Satouchi M. Phase 2 study of nimotuzumab in combination with concurrent chemoradiotherapy in patients with locally advanced non-small-cell lung cancer. Clin Lung Cancer (2021) 22:134–41. doi: 10.1016/j.cllc.2020.12.012 33518480

[51] Qiu B Wang D Li Q Wu Y Guo S Jiang X. Concurrent chemoradiation therapy with or without nimotuzumab in locally advanced squamous cell lung cancer: A phase 2 randomized trial. Int J Radiat Oncol Biol Phys (2021) 111:917–25. doi: 10.1016/j.ijrobp.2021.06.032 34229051

[52] Bradley JD Hu C Komaki RR Masters GA Blumenschein GR Schild SE. Long-term results of NRG oncology RTOG 0617: standard- versus high-dose chemoradiotherapy with or without cetuximab for unresectable stage III non-small-cell lung cancer. J Clin Oncol (2020) 38:706–14. doi: 10.1200/JCO.19.01162 PMC7048161 31841363

[53] Trédaniel J Barlési F Le Péchoux C Lerouge D Pichon É Le Moulec S. Final results of the IFCT-0803 study, a phase II study of cetuximab, pemetrexed, cisplatin, and concurrent radiotherapy in patients with locally advanced, unresectable, stage III, non-squamous, non-small-cell lung cancer. Cancer Radiother (2022) 26:670–7. doi: 10.1016/j.canrad.2021.12.005 35260342

[54] Peng L Wang Y Fei S Wei C Tong F Wu G. The effect of combining Endostar with radiotherapy on blood vessels, tumor-associated macrophages, and T cells in brain metastases of Lewis lung cancer. Transl Lung Cancer Res (2020) 9:745–60. doi: 10.21037/tlcr-20-500 PMC7354151 32676336

[55] Zhu J Chen G Niu K Feng Y Xie L Qin S. Efficacy and safety of recombinant human endostatin during peri-radiotherapy period in advanced non-small-cell lung cancer. Future Oncol (2022) 18:1077–87. doi: 10.2217/fon-2021-1239 34986655

[56] Zhai Y Ma H Hui Z Zhao L Li D Liang J. HELPER study: A phase II trial of continuous infusion of endostar combined with concurrent etoposide plus cisplatin and radiotherapy for treatment of unresectable stage III non-small-cell lung cancer. Radiother Oncol (2019) 131:27–34. doi: 10.1016/j.radonc.2018.10.032 30773184

[57] Honglian M Zhouguang H Fang P Lujun Z Dongming L Yujin X. Different administration routes of recombinant human endostatin combined with concurrent chemoradiotherapy might lead to different efficacy and safety profile in unresectable stage III non-small cell lung cancer: Updated follow-up results from two phase II trials. Thorac Cancer (2020) 11:898–906. doi: 10.1111/1759-7714.13333 32068962 PMC7113061

[58] Chen L Tong F Peng L Huang Y Yin P Feng Y. Efficacy and safety of recombinant human endostatin combined with whole-brain radiation therapy in patients with brain metastases from non-small cell lung cancer. Radiother Oncol (2022) 174:44–51. doi: 10.1016/j.radonc.2022.06.022 35788355

[59] Gao Y Liu P Shi R. Anlotinib as a molecular targeted therapy for tumors. Oncol Lett (2020) 20:1001–14. doi: 10.3892/ol.2020.11685 PMC7377159 32724339

[60] Han D Zhang J Bao Y Liu L Wang P Qian D. Anlotinib enhances the antitumor immunity of radiotherapy by activating cGAS/STING in non-small cell lung cancer. Cell Death Discovery (2022) 8:468. doi: 10.1038/s41420-022-01256-2 36443299 PMC9705441

[61] Wang YX Cheng C Zhuang HQ. The efficacy and safety of Anlotinib combined with stereotactic radiosurgery in the treatment of brain metastases from non-small cell lung cancer. Chin Med J (2022) 102:930–4. doi: 10.3760/cma.j.cn112137-20211013-02262 35385964

[62] He Z Liu J Ma Y Jiang H Cui Z Wang G. Anlotinib combined with cranial radiotherapy for non-small cell lung cancer patients with brain metastasis: A retrospectively, control study. Cancer Manag Res (2021) 13:6101–11. doi: 10.2147/CMAR.S319650 PMC8349551 34377028

[63] Liu J Xu J Ye W Zhong W Zhang X Mao J. Whole-brain radiotherapy combined with anlotinib for multiple brain metastases from non-small cell lung cancer without targetable driver mutation: A single-arm, phase II study. Clin Med Insights Oncol (2022) 16:11795549221079185. doi: 10.1177/11795549221079185 35250325 PMC8891900

[64] Jiang S Zhou Y Zou L Chu L Chu X Ni J. Low- dose Apatinib promotes vascular normalization and hypoxia reduction and sensitizes radiotherapy in lung cancer. Cancer Med (2023) 12:4434–45. doi: 10.1002/cam4.5113 PMC9972072 36065943

[65] Li L Li Y Zou H. A novel role for apatinib in enhancing radiosensitivity in non-small cell lung cancer cells by suppressing the AKT and ERK pathways. PeerJ (2021) 9:e12356. doi: 10.7717/peerj.12356 34760374 PMC8557687

[66] Ren Y Wang SB Zhou L Liu SQ Du LY Li T. Continuous low-dose apatinib combined with WBRT significantly reduces peritumoral edema and enhances the efficacy of symptomatic multiple brain metastases in NSCLC. Technol Cancer Res Treat (2021) 20:15330338211011968. doi: 10.1177/15330338211011968 33955301 PMC8111549

[67] Ma J Pi G Bi J Li Y He H Li Y. Concurrent apatinib and brain radiotherapy in patients with brain metastases from driver mutation-negative non-small-cell lung cancer: study protocol for an open-label randomized controlled trial. Clin Lung Cancer (2021) 22:e211–4. doi: 10.1016/j.cllc.2020.10.007 33187916

